# Prevalence and risk factors of ovine haemonchosis in Kutaber district, north-east Ethiopia

**DOI:** 10.1016/j.vas.2026.100797

**Published:** 2026-08-04

**Authors:** Paulos Gashaw Admass, Assaye Wollelie Fentie, Animaw Andargie Worku, Asmamaw Bihonegn Desale, Teketay Bayleyegn Derso, Yihenew Getahun Ambaw, Zemenu Tsehay Bogale, Assaye Desta Amare

**Affiliations:** a Department of Veterinary Medicine, College of Agricultural Sciences, Woldia University, Woldia, Ethiopia; b Department of Clinical Medicine, School of Vetrinary Medicine, Bahir Dar University, Bahirdar, Ethiopia; c Department of Biomedical and Paraclinical sciences, School of Vetrinary Medicine, Bahir Dar University, Bahirdar, Ethiopia

**Keywords:** Prevalence, *Haemonchus contortus*, Ovine, Kutaber, Risk factors

## Abstract

**Background:**

Ovine haemonchosis is a widespread parasitic disease of sheep in Ethiopia, and understanding its prevalence and associated risk factors in Kutaber district is important for effective control.

**Methods:**

The research was cross-sectional study conducted from December 2023 to May 2024 at kutaber district, North Eastern Ethiopia with the objectives to determine prevalence of ovine haemoncosis and investigating potential associated risk factors. A total of 384 animals were selected by simple random sampling during the study period. parasitological diagnosis was based on coprology and larval culture

**Results:**

Overall prevalence of Haemonchus contortus was found 58.33% in the study area. Prevalence of Haemonchus contortus infection was found significantly higher in animals with poor body condition (76.92%) compared to medium body condition (54.92%)(χ^2^=33.669, OR=2.7, CI=1.65–4.5) and good body condition (31.48%) (*p* < 0. 05, χ^2^=33.669, OR= 7.25, CI=3.5–14.87). Higher prevalence of ovine haemonchosis was recorded in young sheep (62.28%) than in older sheep (56.33%); in female (60.67%) than in male (54.31%). The prevalence of Haemonchus contortus infection was higher among sheep originating from Beshilo Kebele (62.5%); however, the differences were not statistically significant (*p* > 0.05). The difference in prevalences for age, sex and origin was found insignificant (*p* > 0.05).

**Conclusions:**

This study concluded that, body condition was the most important factor where-as age, sex and origins of sheep were found less important factors higher prevalence ovine haemoncosis in this study. Therefore effective strategic treatment like seasonal deworming approach and public awareness creation should be instituted in the study area.

**Abbreviations**
GIN: gastrointestinal nematode
H. contortus: haemonchus contortus
L3: Third Stage larvae
PCV: packed cell volume

## 1 Introduction

Small ruminants, especially sheep, are the backbone of rural livelihoods in Ethiopia and drive a massive portion of the national economy through the production of meat, skin, and wool (Mekuria et al., 2018). Unfortunately, sheep production rarely hits its true potential because flocks are constantly held back by gastrointestinal nematodes (GIN). These internal parasites cause quiet but devastating financial losses, poor growth rates, and widespread mortality across the country's tropical and subtropical zones (Shahi & Rekha, 2023). On a global scale, ovine haemonchosis—caused by the abomasal worm Haemonchus contortus—stands out as the single most destructive parasite limiting sheep farming (Abera, 2018).

The heavy economic toll of H. contortus comes down to its aggressive blood-feeding habits. Both the large immature larvae (L5) and adult worms slice into the lining of the stomach to drink blood, while simultaneously releasing a hemolytic factor that damages the animal's red blood cells (Arsenopoulos et al., 2021). A heavy burden of about 5000 worms can cause a single sheep to lose up to 250 ml of blood every day. This rapid blood loss triggers severe anemia, watery swelling under the jaw ("bottle jaw"), extreme dehydration, and sudden death—often before the owner even realizes the sheep is sick (Flay et al., 2022). To make matters worse, the parasite can go into a state of hibernation (hypobiosis) inside the animal during dry or cold seasons, waiting to trigger explosive outbreaks the moment the weather improves (Yazie, 2015).

Pinpointing this disease in the field is a major diagnostic bottleneck. Standard fecal flotation tests can only spot "strongyle-type" eggs, which look identical across many different species. Because of this, you cannot tell a highly dangerous H. contortus infection apart from a mild, less harmful worm species just by looking at the eggs. Accurate diagnosis requires coprology combined with larval culture and the modified Baermann technique. This allows researchers to hatch the eggs and carefully inspect the third-stage (L₃) larvae under a microscope, identifying them by their distinct head, tail, and sheath structures (Zarlenga et al., 2016).

Managing and controlling these parasites has become an uphill battle. For decades, the livestock sector has relied almost entirely on routine, blanket deworming using standard anthelmintic drugs like albendazole, ivermectin, moxidectin, and levamisole (Adduci et al., 2022). However, this continuous and aggressive use of chemicals has backfired globally, fueling widespread anthelmintic resistance (Calvete et al., 2020). Constant chemical drenching also robs the flock of the chance to naturally build up its own immunity, trapping farmers in a costly cycle of drug dependency and failing treatments. To break this cycle and design sustainable control programs, veterinarians need precise, local data. Across Ethiopia, previous studies show that haemonchosis prevalence fluctuates wildly depending on the region—ranging from a staggering 90.8% in some areas to 26.8% or 46.5% in others (Abebe & Esayas, 2001; Mesele et al., 2014; Mussa, 2023). These numbers prove that parasite threats are highly localized, shifting according to specific microclimates and grazing habits.

Despite extensive research elsewhere in the country, there is absolutely no published data on the prevalence or risk factors of ovine haemonchosis in the Kutaber District. This local information gap leaves smallholders and agricultural extension officers completely in the dark, making it impossible to plan targeted treatments or surveillance. Therefore, this study was designed to determine the actual prevalence of ovine haemonchosis and uncover the key risk factors driving it in Kutaber District, providing the baseline evidence needed to safeguard local sheep production.

Given the global crisis of widespread anthelmintic resistance, sustainable parasite control has shifted away from intensive, blanket drenching toward Integrated Parasite Management (IPM) frameworks (Hoste et al., 2021). Modern international literature heavily emphasizes Targeted Selective Treatment (TST) protocols, such as the FAMACHA eye color chart system, which identifies and treats only the specific individuals showing clinical signs of severe anemia rather than the entire flock. This approach actively preserves a population of "refugia"—parasites not exposed to the drug—which slows down the selection pressure for resistance alleles and prolongs chemical efficacy (Van Wyk, 2022). Implementing these integrated strategies alongside rotational grazing and selective breeding is vital for sustainable livestock production, but success depends entirely on having accurate, area-specific baseline data on local parasite dynamics. The aim of the study was to determine the prevalence of ovine haemonchosis and identify associated risk factors like age, sex, and body condition in sheep from the Kutaber District, North-East Ethiopia. Therefore, this study was imperative to fill the information gap in the study was to determine overall prevalence of ovine haemonchosis and to asses influence associated risk factors on the occurrence of ovine haemonchosis.

## 2 Materials and methods

### 2.1 Study area

**Fig. 1:**
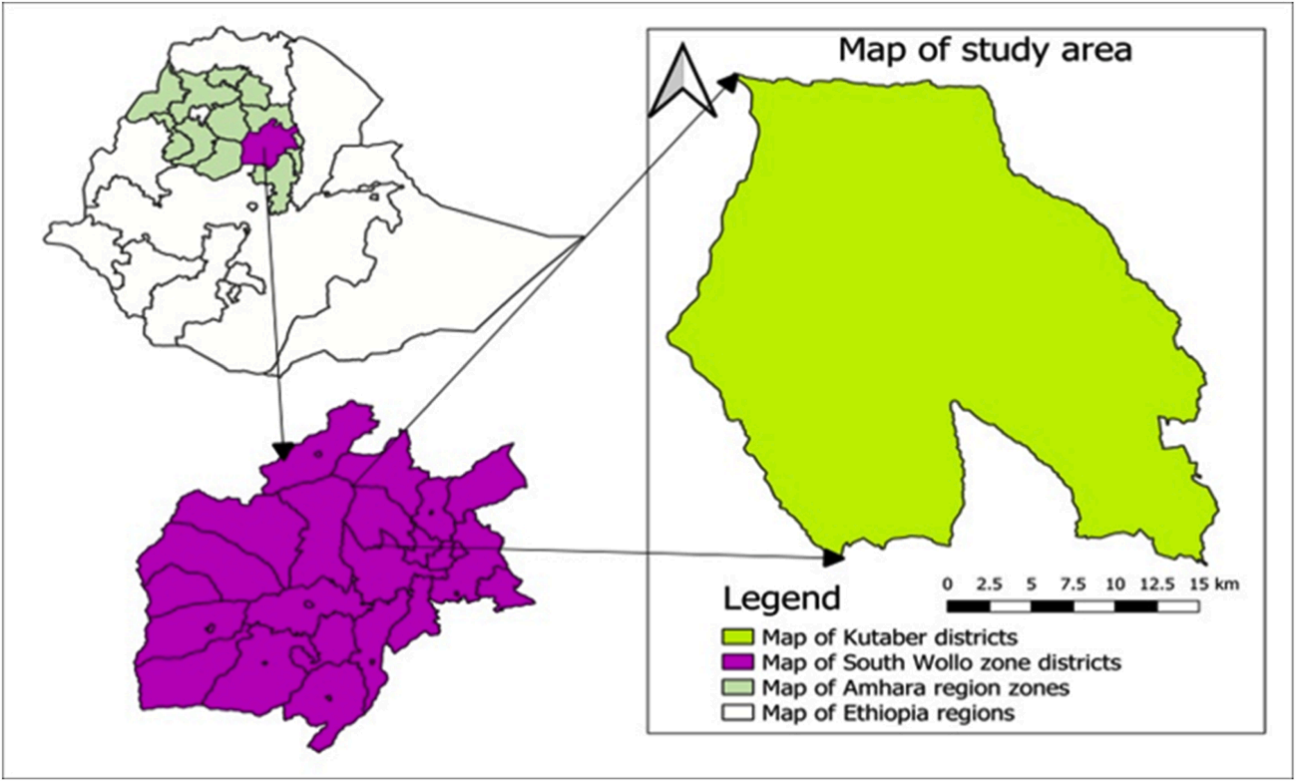
Map of study area. (Source: Q-GIS and Ethiopia boundaries shape file).

Kutaber district as indicated in Fig. 1 is located at 110 12′ 36″ −110 18′ 36″ N latitude and 390 31′12″-390 34′12″ E longitude. Kutaber area poses highland and lowland areas. The average minimum and maximum rainfall ranges between 500 and 955 ml in short and long rainy season. The average annual temperature is 22 °C (Kado, 2018). Mixed agriculture is the main occupation of the population of the area. The major livestock reared in the area are sheep, goat, cattle, poultry and equine. According to statistical data, Kutaber Woreda has livestock population of 69 720 cattle, 65 727sheep, 53 304goats, 18 005 Equines and 104 737 chickens (Klrdo, 2018). Sheep farming and management practices in the Kutaber district of Ethiopia are predominantly characterized by a traditional, low-input, extensive system, often integrated with mixed crop-livestock farming. As a highland area with a high sheep population density, sheep are a crucial source of income and social security(Klrdo, 2018).

### 2.2 Study population

The study population consisted of sheep of different ages and sexes, managed under predominantly extensive grazing systems, with some variation in management practices across the study area. Local breeds of sheep by considering different age groups (<1year, young and >2-years, adult), sex group (male and female) and dental/chronological rule was used to detemine age, Body condition was assessed using a standardized 1-to-5 body condition scoring system and subsequently categorized into three groups as poor, medium, and good for statistical analysis. The scoring was based on established physical assessment criteria to ensure consistency among animals and origin of animal in the study area were populations for this study.

### 2.3 Study design and sample size determination

A cross-sectional study was conducted from December 2023 to May 2024 to determine the prevalence of ovine haemoncosis and its associated risk factors in the study area. Sex, age, body condition and origin considered as independent variables. To determine sample size, prevalence rate of 50% was taken in to consideration since there was no research done on ovine haemonchosis. The desired sample size for the study was calculated by using formula given by Thrusfield (2005) with desired precision of 5% at confidence level of 95%.

$$N=\frac{1.96^{2\;}\times \mathrm{Pexp}\left(1-\mathrm{Pexp}\right)}{d^{2}}$$

Where: N = required sample size; Pesp = estimated population (50%); d = desire absolute precision (5%). Therefore, based on the formula total of 384 animals were selected for this study.

### 2.4 Sampling method

To achieve the objectives of this study, we used a multi-stage sampling design across the Kutaber district. First, we randomly selected four kebeles: Alansha, Beshilo, Boru Meda, and Kutaber Town (Kebele 01). Next, we used simple random sampling to select individual smallholder households within each kebele, using registration lists from the local agricultural offices as our guide. Because flock sizes vary in the field, we sampled multiple eligible sheep per household (ranging from 1 to 5 animals depending on what the owner had available). This means the number of sampled animals naturally varied between households. In total, we examined 384 local breed sheep for ovine haemonchosis. During visits, we recorded individual animal data along with household IDs and geographic origins.

### 2.5 Sample collection, handling and transport

Fecal material were collected directly from rectum of each sheep to sample collecting bottle (screw capped bottle) directly from the rectum of ovine, and each samples were given a labeled considering date, age, sex, origin and body condition. All collected samples were transport to Wollo University, Parasitology Laboratory by using an ice box and stored at (4 °C) refrigeration until processing.

### 2.6 Laboratory examination procedures

Fecal samples were examined using the saturated salt solution flotation technique (specific gravity 1.20) to detect strongyle-type nematode eggs. Briefly, fecal material was mixed thoroughly with saturated salt solution, filtered, and examined microscopically for the presence of nematode eggs. Eggs with an ellipsoidal shape, thin and smooth shell, and morula-stage blastomeres were identified as strongyle-type eggs based on their morphological characteristics (Kumsa, 2007). Because strongyle-type eggs cannot be reliably differentiated to genus level by routine fecal examination, each sheep that was positive for strongyle-type eggs was subjected to individual fecal culture for further identification of the infecting nematode.

For fecal culture, the strongyle-positive fecal sample from each individual sheep was thoroughly mixed, kept moist and crumbly, and transferred into a Petri dish. The cultures were incubated at approximately 27 °C for 7–10 days under moist and well-aerated conditions to allow the eggs to hatch and develop into third-stage larvae (L3). During incubation, the cultures were mixed periodically and kept sufficiently moist. After incubation, the L3 larvae were recovered using the modified Baermann technique.

The recovered L3 larvae were examined microscopically and identified based on their morphological features, particularly the characteristics of the head and tail. The presence, relative proportion, and length of the sheath tail extension (STE) were also considered during identification. Haemonchus contortus L3 larvae were distinguished from other strongyle-type larvae based on their characteristic morphological features, following the criteria described by Zarlenga et al. (2016). Therefore, strongyle-type eggs detected by fecal flotation were used for initial screening, while confirmation of H. contortus infection was based on the identification of H. contortus L3 larvae from the individual fecal culture of each strongyle-positive sheep.

### 2.7 Data analysis

All collected and coded data were entered in to Microsoft excel and analyzed by using IBM-SPSS version 20.0. Descriptive statistics were used to summarize the data with regard to frequencies and percentage. Tables, Charts and Graphs were used to present the data. The association of the different factors with prevalence of the disease was analyzed using chi- square (X ^2^) test, 95% confidence interval and *p* < 0.05 confidence levels were used to determine the significant level.

## 3 Results

As indicated in the (Table 1) below; out of total 384 sheep examined, 224 were found to be positive for haemonchus contortus parasite. Overall prevalence of ovine haemoncosis was found 58.33%. A higher prevalence of ovine haemoncosis was observed in young (62.28%) as compared to adult (56.66%); in female (60.67%) as compared to male (54.31%). But the difference for age (χ² = 1.039, *p* > 0.05) and sex (χ² = 1.107, *p* > 0.05) was found insignificant.

**Table 1:** Associated risk factor Haemonchus Contortus.

Risk factors	Category	N	Number of Positive%	X^2^	*p*-value	OR(CI)
Age	Young	114	71(62.28%)	1.039	0.308	1.26(0.81–1.98)
	Adult	270	153(56.33%)			
Sex	Male	116	63(54.31%)	1.107	0.293	1.26(0.815–1.96)
	Female	268	161 (60.67%)			
Body condition	Poor	117	90 (76.92%)			Ref.
	Medium	213	117(54.92%)	33.66	0.000	2.7(1.65–4.5)
	Good	54	17(31.48%)			7.25(3.5–14.87)
Origin	Alansha	96	53(55.20%)	1.114	0.774	Ref.
	Beshilo	96	60(62.50%)			1.35(0.76–2.410
	Kutaber	96	56(58.33%)			1.14(0.64–2)
	Borumeda	96	55(57.29%)			1.1(0.615–1.925)
Overall		**384**	**58.33%**			

Ref. =Reference, X^2^=Chi-square, OR= Odds ratio, CI= Confidence Interval, N = number examined.

Body condition score was significantly associated with *H. contortus* prevalence (χ² = 33.669, *p* < 0.05). Regarding origin of the animal the difference was found insignificant (χ² = 1.114; *p* > 0.05).

## 4 Discussion

*Haemonchus contortus* is endo-parasite with the greatest pathogenic and economic importance in small ruminants. It is important to determine the prevalence of ovine haemonchosis to recommend the most beneficial and economically acceptable control measures. The determination of the risk factors associated with parasite occurrence can be used to design an effective control strategy (Odoi et al., 2007).

This study was revealed with overall prevalence of 58.33% ovine haemoncosis from total of 384 sheep Coprological examination. This result indicated that there is a very high level of parasitic infestation in the study area. Further possible reason for the high prevalence of haemonchosis in the study area may be due to the fact that sheep are free grazer with the high stocking density, where large numbers of animals graze together throughout the year in communal grazing land, inadequate nutritional status and lack of community awareness regarding to the parasites economic impact. This result is in line with prevalences of ovine haemonchosis (56.25%) from Bihar Dar Municipal Abattoir by Moges et al. (2017); 62% from Jimma town by Bekuma and Dufera (2019); 68.75% from Jimma town municipal Habte and Ibrahim (2018) and 69.6% from Debrazeit ELFORA export abattoir by Abdo et al. (2017).

The current finding was found lower than prealences of ovine haemoncosis 90.8% repored by Abebe and Esayas (2001); prevalence of 90.4% from Haramaya municipal abattoir, eastern Hararge, Ethiopia by who reported by Argaw et al. (2014); 80.21% from Gonder town by Fentahun and Luke (2012), 83.4% from Rwanda reported by Mushonga et al. (2018), and 80.64% from Pakistan by Asif et al. (2008). This finding was also found lower than prevalence of ovine haemoncosis 26.8% from Mekelle Abergelle export abattoir by Mesele et al. (2014); 33.1% from Jimma municipal abattoir by Abera (2018); 46.1% from Mitto district, silte zone prevalence by Mussa (2023). The observed variation in prevalence of haemonchosis with in the country and outside the country might be due to the differences in variety of factors such as sample size, sampling technique, husbandry practices, environmental factors, host age, breeding status, grazing habits, method of examination, level of community awareness, standard of management and habits of anthelmintic usage which influences the development, distribution and survival of the parasite.

Age wise distribution of ovine haemonchosis indicated that, younger animals were with slightly higher prevalence (62.28%) compared to adult(56.33%) but the difference was statistically insignificant (*p* > 0.05, χ² = 1.039, CI= 0.81–1.89) (tabel 1). This finding was related with previous findings which were reported by (Gonfa Shankute et al., 2013) and (Mesele et al., 2014) in Ethiopia. Resulted slight difference between young and adult might be due to low immunity of resistance or greater susceptibility of young animal and young ovine have not been exposed earlier in the infection. Also as stated by (Blackie, 2014) graduall increase in exposure to parasitic infection, the immune system of host animals builds up especially against Haemonchus contortus and age resistance develops. The current finding were disagrees with the previous work by (Habte & Ibrahim, 2018; Tesfaye, 2021) who observed higher prevalence of ovine haemoncosis in adult compared to young.

Regarage the sex difference; female animals were found with slightly higher prevalence 60.67% compared to male 54.31% but, difference not significant (*p* > 0.05). The existed slight difference may be due to the reason that females are more exposed to stress than males in different condition such as during lactation; lactating in sheep and therefore females are weaker and less able to resist the effects of parasites on their body, and ewes and does in late gestation, and for a short period after parturition lose much of their resistance to parasites due to hormonal and photo-period effects as also stated by Moges et al. (2017). This finding goeses inline with findings in ethiopia observed by (Mussa, 2023) and contradicted with findings of Mengist et al. (2014) and Mideksa et al. (2016).

Body condition score was significantly associated with *H. contortus* prevalence significantly higher prevalence (76.92%) was detected in animals with poor body condition (χ^2^=33.669, OR=2.7, CI=1.65–4.5) and good body condition (31.48%) (*p* < 0. 05, χ^2^=33.669, OR= 7.25, CI=3.5–14.87) (tabel 1). Body condition is a key factor in ovine haemonchosis because it reflects a sheep's nutritional status and underlying immune function, making animals in poor body condition more vulnerable to infection and disease severity. Poorly conditioned sheep have compromised immunity, which allows the Haemonchus contortus parasite to establish, develop, and cause greater harm, leading to higher infestation rates and a worse prognosis. Existed significantly highest prevalence rate recorded in poor body conditioned sheep had indicated that the change in appetite for infected sheep’s attributed to hormonal changes like that of gastrin level which affects feed intake and due to weight loss that leads to decline in immune status of the animal as it was also stated by Hoste (2001). This result goeses agrees with findings by Moges et al. (2017) whom observed higher prevalence in poor body conditioned animals compared to animals with medium and good body conditions. This might also be due o the reason that animals with good body condition have good immunity that inhibits the fecundity of the parasites as it was also mentioned by (Farooq and Abera, 2018). However present finding ccontradicts with the research reported by Abebe et al., who found higher prevalence of ovine haemonchosis in animals with good body condition compared to medium and poor body condition. This might be due to different factors like seasonal change of feed and difference in Poor management system.

The prevalence of *H. contortus* in ovine that originated from different sites of the study area indicated different prevalence. The prevalence of haemonchosis was higher in those ovine sampled from Beshilo with the rate of 62.50% followed by sheep originated from Kutaber town (kebele 01), Boru meda and Alansha with the rates of 58.33%,57.29% and 55.20%, respectively. But differences between the prevalence of haemonchosis were satistically insignificant (*p* > 0.05). The relatively high occurrence of Haemonchus contortus in the study area may be linked to the widespread practice of communal grazing, where many sheep graze together on the same pasture. This can increase contamination of grazing areas with parasite eggs and larvae, making transmission easier among animals. Poor nutritional status may further increase susceptibility by weakening the animals’ ability to respond to infection. In addition, the local climate and environmental conditions in Kutaber may provide suitable conditions for the development and survival of H. contortus larvae on pasture. These factors, together with the existing grazing practices, may help explain the continued transmission of haemonchosis among sheep in the area. Controlling ovine haemonchosis involves integrated approaches, including strategic and targeted anthelmintic use, careful grazing management to minimize larval exposure, improving animal husbandry through balanced nutrition, maintaining animal health through vaccination and resistance management, and implementing continuous monitoring and diagnosis through fecal egg counts and surveillance.

### 4.1 Study limitation

This study has some limitations that should be considered when interpreting the findings. Since the study used a cross-sectional design, it could not establish a causal relationship between the identified risk factors(body condition) and ovine haemonchosis. Although Haemonchus contortus L3 larvae were identified morphologically from individual fecal cultures, molecular methods were not used for species confirmation. In addition, factors such as previous anthelmintic use and grazing history were not assessed in detail and may have affected the observed prevalence. This study was conducted during a single study period, so seasonal variations in the prevalence of H. contortus may not have been fully captured. This study used qualitative detection of Haemonchus contortus infection and did not include fecal egg counts (EPG) or direct worm burden measurements; therefore, the intensity and severity of infection could not be assessed.

## 5 Conclusion and recommendations

Ovine haemonchosis caused by the blood-feeding nematode Haemonchus contortus is an important parasitic disease that can negatively affect sheep health and productivity. In this study, body condition was significantly associated with the occurrence of H. contortus infection, with sheep in poorer body condition being more affected. This finding highlights the importance of maintaining good nutritional status and overall body condition as part of haemonchosis control. In contrast, age, sex, and place of origin were not significantly associated with infection, indicating that these factors had a limited influence on the prevalence observed in the study area. Therefore, control efforts should focus on improving nutrition and husbandry practices, together with appropriate and strategic use of anthelmintics and regular monitoring of infection. The findings of this study provide useful local evidence for improving the prevention and control of ovine haemonchosis in the study area. Based on conclusions above the following recommendations are forwarded:

1. There should be rotational grazing of pasture land to control Haemonchus parasites and maintaining the stocking rate level avoids consequent pasture contamination.
2. Regular deworming should be implemented to decrease the population of the parasites in the small ruminant.
3. Awareness creation of community with regards of economic significances and risk factors of Haemonchosis on small ruminants should be provided.
4. Government should develop policies for integrtated parasitic control strategies, disease surveillance, and farmer education to ensure a sustainable and profitable sheep sector.

## Declarations

### Ethical statement

Ethical approval for animal sampling was obtained from the Woldia University Research Ethics Committee. All procedures followed institutional and national guidelines for animal welfare. Farmer consent was obtained prior to sampling.

### AI statement

During the preparation of this work the author(s) used Microsoft copilot in order to rewrite the cover letter and manuscript. After using this tool/service, the author(s) reviewed and edited the content as needed and take(s) full responsibility for the content of the published article.

## Funding information

This research received no specific external funding.

## Consent for publication

Not applicable.

## Informed consent statement

Not applicable.

## CRediT authorship contribution statement

**Paulos Gashaw Admass:** Writing – review & editing, Writing – original draft, Methodology, Investigation, Formal analysis, Data curation, Conceptualization. **Assaye Wollelie Fentie:** Writing – review & editing, Writing – original draft, Validation, Software. **Animaw Andargie Worku:** Writing – review & editing, Writing – original draft, Validation, Data curation. **Asmamaw Bihonegn Desale:** Writing – review & editing, Methodology, Funding acquisition, Data curation. **Teketay Bayleyegn Derso:** Writing – review & editing, Writing – original draft, Methodology, Data curation. **Yihenew Getahun Ambaw:** Writing – original draft, Validation, Software, Data curation. **Zemenu Tsehay Bogale:** Writing – original draft, Validation, Formal analysis, Data curation. **Assaye Desta Amare:** Writing – review & editing, Writing – original draft, Visualization, Supervision, Resources, Project administration, Methodology, Investigation, Funding acquisition, Formal analysis, Conceptualization.

## Declaration of competing interest

I confirm that all authors of the manuscript have no conflict of interests to declare. I confirm that the manuscript is the authors' original work and the manuscript has not received prior publication and is not under consideration for publication elsewhere. I declare that I shall not submit the paper for publication in any other Journal or Magazine till the decision is made by journal editors.

## Figures and Tables

**Annex 1:**
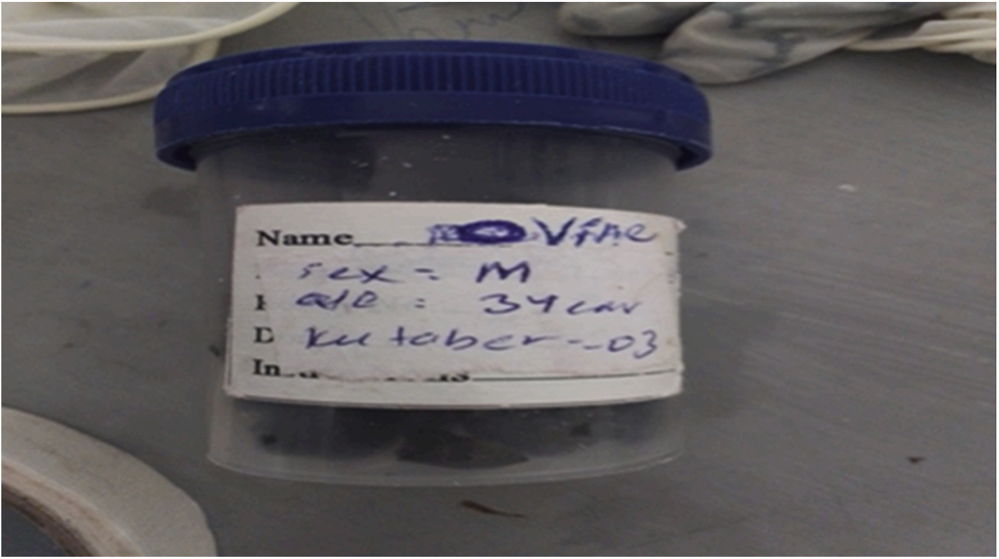
Sample collection procedure and labeling during my study period.

**Annex 2:**
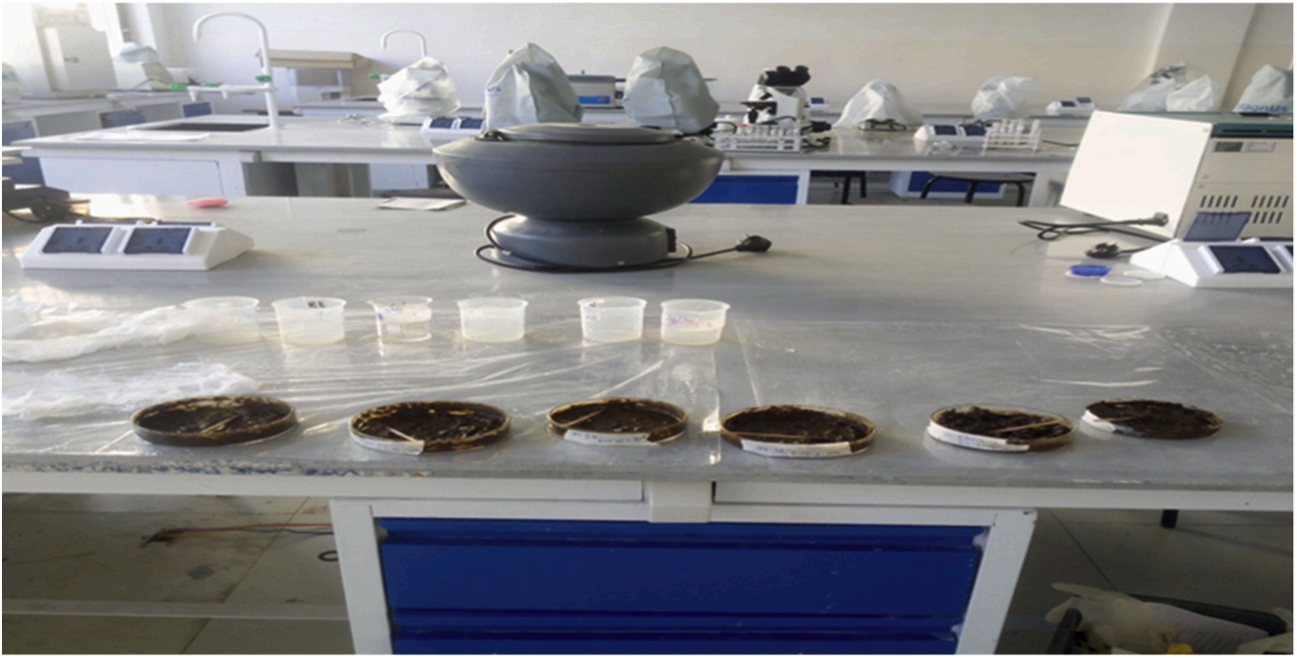
Faecal culturing process to identify *Haemonchus* larvae.

**Annex 3:**
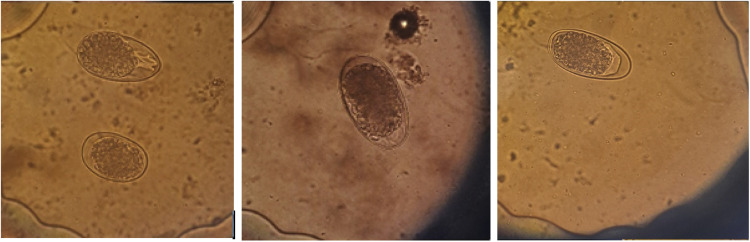
Strongyle type egg found during study area by flotation technique.

**Annex 4:**
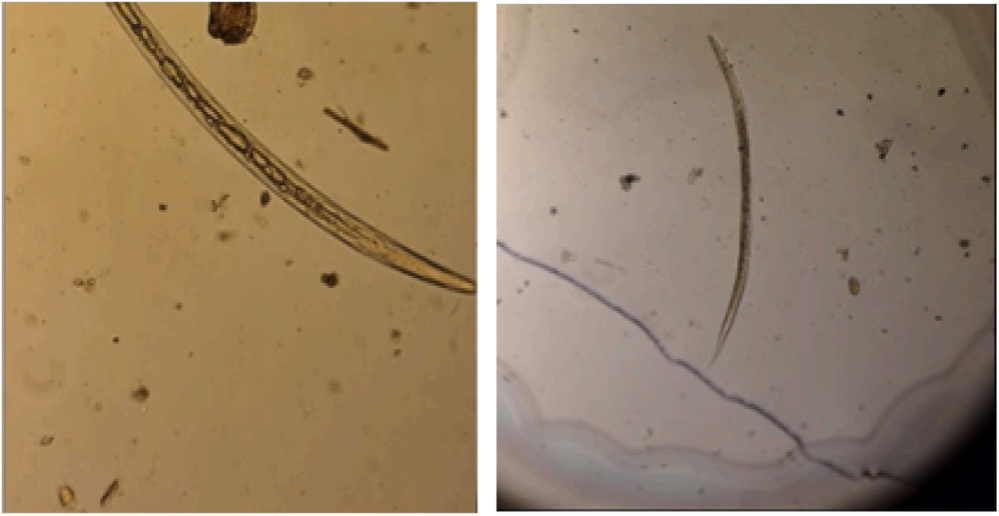
Infective larvae (*L3)* of *H contortus* in young male sheep from Boru meda.

**Annex 5:**
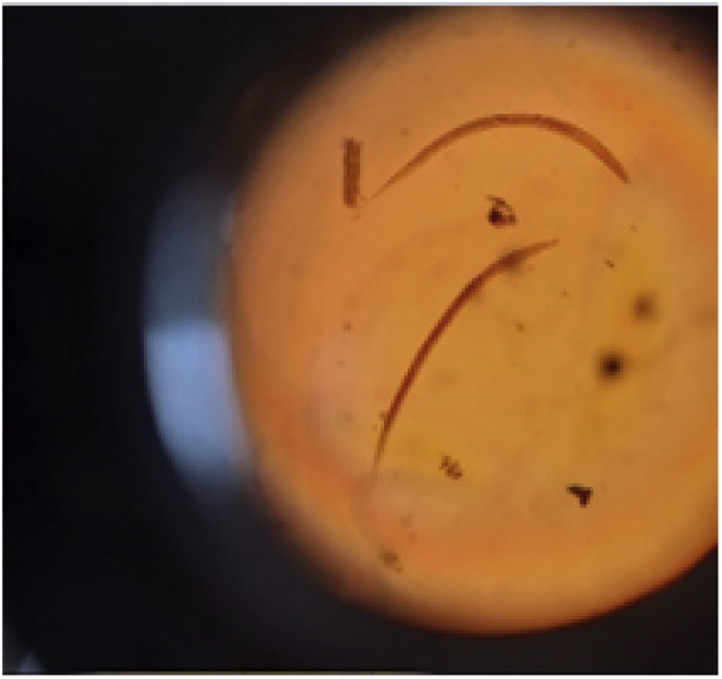
L3 complex nematodes found during study period in young female sheep in Beshilo kebeles.

## Data Availability

The datasets used and/or analyses during the current study are available from the corresponding author on reasonable request.

## List of annexies

Annex 1; Annex 2; Annex 3; Annex 4; Annex 5.
